# 233 Acquisition Rates and Risk Factors for Carbapenem-Resistant Acinetobacter baumannii in an Emergency ICU: A Case-Control Study

**DOI:** 10.1017/ash.2026.10609

**Published:** 2026-06-23

**Authors:** Akiho Iizawa, Mckenzi King, Laura Rusie, Ellen Gough, Sarah Sansom, Alison Peterson, Lisa Duffner, Nidhi Undevia, Michelle Funk, Alyse Kittner, Divya Ramachandran, Thomas Roome, christy zelinski, Mabel Frias, Mary Hayden, Michael Lin

**Affiliations:** 1 Rush University Medical Center; 2 Rush University; 3 RML Specialty Hospital; 4 Chicago Department of Public Health; 5 Cook County Department of Public Health

## Abstract

**Background:** Previous studies demonstrated that the addition of anterior nares to axilla/groin skin testing substantially improved the detection of Candidozyma auris colonization (Proctor et al., Nature Medicine, 2021; Sansom et al., CID, 2024), but these observations were limited by small sample sizes and reflected mostly nursing home residents. We aimed to assess the benefit of adding nares testing to axilla/groin testing among hospitalized patients participating in C. auris point prevalence surveys (PPSs). Methods We conducted a prospective observational study at three long-term acute care hospitals (LTACHs) in the Chicago region, one of which also included distinct inpatient rehabilitation floors. Facilities participated in quarterly public health-led C. auris PPSs from May 2024 to October 2025. Patients underwent swab sampling at two sites: composite bilateral axillae/groin (swab 1) and bilateral anterior nares (swab 2). Samples were processed at a central laboratory for C. auris detection by PCR using a validated assay with enzymatic preprocessing and automated DNA extraction. Primary analyses were restricted to patients with results available from both sampling sites; a positive C. auris test from either body site was considered the reference standard. Analyses were performed using R v4.5.2 (www.r-project.org) and Stata/SE v18.0 (Stata Corp., College Station, TX). Results Across 18 PPSs, 769 of 867 (89%) eligible patients participated. The overall C. auris prevalence was 46% (317/685) among LTACH patients and 15% (13/84) among rehabilitation patients. Among all patients who tested positive for C. auris, 64% were in Contact Precautions for any reason at time of PPS and 34% were previously known to be C. auris colonized. After excluding 16 patients who were missing a body site specimen, 753 patients were eligible for analysis (670 LTACH, 83 rehabilitation). Among 320 patients with C. auris detected at any body site, 301 were detected by axilla/groin screening (94% sensitivity; 95% confidence interval 91% to 96%) and 234 (73%) tested positive at the anterior nares for C. auris. Inclusion of nares swabbing identified 19 additional patients who would have been missed by axilla/groin screening alone, corresponding to an incremental increase of 6%. Conclusion Among LTACH and rehabilitation patients, C. auris colonization was common and PPSs identified a substantial number of patients not previously known to be colonized. The incremental benefit of adding nares screening was modest compared to axilla/groin screening alone. Testing more body sites can identify more C. auris colonized patients, but facilities should balance benefit versus cost of expanded screening approaches.

